# Editorial: Clinical, Molecular and Adverse Responses to B-Cell Therapies in Autoimmune Disease

**DOI:** 10.3389/fimmu.2022.962088

**Published:** 2022-07-06

**Authors:** Savino Sciascia, Ioannis Parodis, Mohammed Yousuf Karim

**Affiliations:** ^1^ University Center of Excellence on Nephrologic, Rheumatologic and Rare Diseases (ERK-net, ERN-Reconnect and RITA-ERN Member) with Nephrology and Dialysis Unit and Center of Immuno-Rheumatology and Rare Diseases (CMID), Coordinating Center of the Interregional Network for Rare Diseases of Piedmont and Aosta Valley, San Giovanni Bosco Hub Hospital, Turin, Italy; ^2^ Department of Clinical and Biological Sciences, University of Turin, Turin, Italy; ^3^ Division of Rheumatology, Department of Medicine Solna, Karolinska Institutet and Karolinska University Hospital, Stockholm, Sweden; ^4^ Department of Rheumatology, Faculty of Medicine and Health, Örebro University, Örebro, Sweden; ^5^ Department of Pathology, Sidra Medicine, Doha, Qatar

**Keywords:** B-cell, rituximab, belimumab, hypogammaglobulinemia, autoimmunity, allergy, adverse, neutropenia

In this Research Topic, we highlighted advances and addressed knowledge gaps in prediction, mechanisms and management of adverse events and efficacy of B-cell targeted therapies (BCTT) in autoimmune disease. BCTT were introduced in 1997 for treatment of lymphoma, and subsequently have become an important treatment option for a wide range of autoimmune diseases, particularly autoimmune rheumatic diseases (AIRD), including for the management of severe patients. BCTT include B-cell depleting drugs (BCDT) targeting CD20 (e.g. rituximab-RTX), CD22 (e.g. epratuzumab), and CD19 (e.g. MEDI-551); and drugs interfering with B-cell survival factors, such as belimumab. Indeed, the latter is one of only three new therapies for patients with systemic lupus erythematosus (SLE) or lupus nephritis (LN) to have received a license from the US Food and Drug Administration over the last 60 years. Several studies have tested the combination of different BCTT, e.g. RTX and belimumab. The BLISS-BELIEVE and CALIBRATE clinical trials reported negative efficacy results for add-on RTX compared with belimumab alone (SLE) or add-on belimumab compared with RTX and cyclophosphamide alone (LN), respectively. However, the BEATLupus study showed that add-on belimumab was superior over RTX alone in prolonging the time to severe SLE flare and in reducing anti-dsDNA antibody levels (1–3). Besides, as incomplete peripheral blood B-cell depletion might be associated with the inability to reduce tubulointerstitial lymphoid aggregates in the kidney and be responsible for inadequate response to treatment (4) a short-term intensified BCTT (5, 6) consisting of a combination therapy of RTX and cyclophosphamide given at sub-immunosuppressive doses aimed at potentiating the B cell depleting effects of RTX was developed and showed effective results even in the long term (without immunosuppressive maintenance therapies) (5, 6).

Clinical use of BCTT is expanding: the BCDT agent RTX is now approved in ANCA-associated vasculitis (AAV), rheumatoid arthritis (RA), and pemphigus, while ocrelizumab (relapsing-remitting and primary progressive) and ofatumumab (relapsing-remitting) are approved in multiple sclerosis. BCTT are used off-label in lupus, membranous nephropathy, Sjögren’s syndrome and certain autoimmune neurological disorders. In this Research Topic, Wang et al. reported clinical benefit of lower dose RTX in 19/26 (73.1%) patients with severe autoimmune encephalitis, with add-on bortezomib (proteasome inhibitor targeting plasma cells) in the remaining refractory 7/26 patients. Walhelm et al. observed favourable results using bortezomib in a nationwide Swedish study of 12 patients with refractory SLE and/or lupus nephritis. Cui et al. described a case report of belimumab treatment for anti-SRP-associated immune-mediated necrotizing myopathy.

While evidence supporting BCDT efficacy in several autoimmune conditions is increasing, current monitoring of BCTT remains rudimentary, and there are major opportunities to develop predictive biomarkers and immunological monitoring for both efficacy and adverse events. From a *post-hoc* analysis of the major phase III belimumab SLE trials, Parodis et al. noted that early patterns in particular B-cell subsets following standard therapy with or without add-on belimumab might predict future SLE flares. Rapid memory B-cell (MBC) expansion may predict sustained treatment response when followed by a subsequent reduction, while no return or delayed MBC increase may predict disease flare. Arnold et al. proposed a personalized retreatment approach in AAV patients based on clinical assessment using the Birmingham Vasculitis Activity score or B-cell markers. They suggested that all BCTT-treated patients should receive concomitant oral immunosuppression, with further BCTT at 6 months in patients with incomplete clinical response or absent naïve B-cells. In pemphigus treated with RTX, Hebert et al. noted an increase in BAFF levels and BAFF-R on B-cells, in contrast to patients receiving corticosteroids alone, in whom BAFF-R was unchanged. Li et al. undertook a cluster analysis of B-cell subsets in IgG4-related disease, stratifying the patients into 3 subgroups: subgroup 1 with low MBC and normal Breg, subgroup 2 with high MBC and low Breg, and subgroup 3 with high plasmablasts and low naive B-cells. This has potential treatment implications as subgroup 2 and 3 patients were overall more treatment-resistant.

Initially, certain adverse effects of BCTT, such as hypogammaglobulinaemia appear to have been underestimated (7). This may have related to various factors, including the short duration and limited number of treatment cycles in early reports. Conversely, progressive multifocal leukoencephalopathy was perhaps over-estimated due to a number of early cases, and the severity of this condition. Studying adverse events cannot be approached in isolation. As we treat patients holistically, we recognize the need to study toxicity in the context of efficacy. Profound and prolonged B-cell depletion may induce clinical remission, but result in sustained hypogammaglobulinaemia in a proportion of patients (8).

Other important adverse effects include neutropenia, hepatitis B reactivation, allergy/infusion reactions, serum sickness, human anti-chimeric antibody responses, and primary or secondary non-response (9–11). There is a clinical need to improve selection of patients being prescribed BCTT based on their likelihood to respond or experience specific adverse events. We need to understand the role of early intervention should such adverse events occur. Tieu et al. reported on a large prospective BCTT cohort of over 400 autoimmune disease patients (Jayne D, personal communication) with long-term follow-up in Cambridge, UK. Of 142 patients (101 AAV, 18 SLE, 23 other) developing persistent hypogammaglobulinemia, 29 (20.4%) required immunoglobulin replacement therapy (IGRT), with consequent reduction in infection risk. In contrast, an Austrian study of 144 autoimmune renal disease patients by Odler et al. reported hypogammaglobulinemia in 58.5% of the patients, but this was not associated with serious infections (SI). Impaired renal function, lower BMI, nephritic glomerular disease treated with corticosteroids, were factors associated with SI. These contrasting conclusions with respect to clinical significance and infection risk may relate to several factors: underlying disease (risk appears higher in AAV); duration of follow-up; definition of hypogammaglobulinemia; cumulative dose of BCTT; cumulative dose and concomitant use of other immunosuppressive agents. This also illustrates the need to recognize that most BCTT-related hypogammaglobulinemia is minor/transient, but that in a significant minority, recurrent/severe infections and persistent hypogammaglobulinemia may justify IGRT (12).

Although RTX is the anti-CD20 agent for which most experience exists, there are several second and third-generation anti-CD20 agents which have been studied in autoimmune disease. Here, Kaegi et al. reported a systematic review of the efficacy and safety of these drugs, including obinutuzumab, ocrelizumab, ofatumumab, ublituximab, and veltuzumab. In a case series of phospholipase A2 receptor (PLA2-R)-associated membranous nephropathy, obinutuzumab showed promising results. Ofatumumab showed promising results in AAV, SLE, and RA, but mixed results in PLA2-R-associated membranous nephropathy.

Patients may be unable to tolerate BCTT due to infusion reactions, development of major allergic responses/anaphylaxis, induction of human anti-chimeric antibodies (HACA). This can lead to a clinical management quandary, for example if the patient’s disease is responding well to the particular BCTT. Aun et al. reported successful desensitization of a multiple sclerosis patient who experienced an allergic reaction during the first infusion of ocrelizumab.

During the COVID-19 pandemic, it has emerged that autoimmune disease patients on immunosuppression may not respond optimally to COVID-19 vaccination – most clearly demonstrated for RTX treatment (13). Here, Stefanski et al. assessed COVID-19 vaccine responses in 15 AIRD patients treated with RTX. In vaccine responders, most B-cells were naïve and transitional, while the B-cell profile in non-responders included mainly plasmablasts and CD27^-^IgD^-^ double negative B-cells. The authors suggested that a significant repopulation of the naive B-cell compartment was positively associated while B-cell exhaustion markers (upregulation of CD95 and loss of CD21) were inversely associated with vaccine response (Stefanski et al.).

From the publications in this Research Topic, we thank the contributing authors for demonstrating the progress of BCTT use in autoimmune disease, with expansion regarding the range of diseases, choice of agents, and studies aiming at optimizing efficacy and safety. Future work will build on this progress, in order to attain multiple ambitions: personalization of BCTT in autoimmune disease; identification of appropriate biomarkers; minimization of infectious complications; and prediction of patients at highest risk of specific side-effects.

## Author Contributions

All authors listed have made a substantial, direct, and intellectual contribution to the work and approved it for publication.

## Conflict of Interest

IP has received research funding and/or honoraria from Amgen, AstraZeneca, Aurinia Pharmaceuticals, Elli Lilly and Company, Gilead Sciences, GlaxoSmithKline,Janssen Pharmaceuticals, Novartis and F. Hoffmann-La Roche AG.

The remaining authors declare that the research was conducted in the absence of any commercial or financial relationships that could be construed as a potential conflict of interest.

## Publisher’s Note

All claims expressed in this article are solely those of the authors and do not necessarily represent those of their affiliated organizations, or those of the publisher, the editors and the reviewers. Any product that may be evaluated in this article, or claim that may be made by its manufacturer, is not guaranteed or endorsed by the publisher.
